# A Novel Interaction between FLICE-Associated Huge Protein (FLASH) and E2A Regulates Cell Proliferation and Cellular Senescence via Tumor Necrosis Factor (TNF)-Alpha-p21WAF1/CIP1 Axis

**DOI:** 10.1371/journal.pone.0133205

**Published:** 2015-07-24

**Authors:** Takahiro Hirano, Taichi Murakami, Hiroyuki Ono, Akiko Sakurai, Tatsuya Tominaga, Toshikazu Takahashi, Kojiro Nagai, Toshio Doi, Hideharu Abe

**Affiliations:** Department of Nephrology, Institute of Health Biosciences, University of Tokushima Graduate School, Tokushima, Japan; Roswell Park Cancer Institute, UNITED STATES

## Abstract

Dysregulation of the cell proliferation has been implicated in the pathophysiology of a number of diseases. Cellular senescence limits proliferation of cancer cells, preventing tumorigenesis and restricting tissue damage. However, the role of cellular senescence in proliferative nephritis has not been determined. The proliferative peak in experimental rat nephritis coincided with a peak in E2A expression in the glomeruli. Meanwhile, E12 (an E2A-encoded transcription factor) did not promote proliferation of Mesangial cells (MCs) by itself. We identified caspase-8-binding protein FLICE-associated huge protein (FLASH) as a novel E2A-binding partner by using a yeast two-hybrid screening. Knockdown of FLASH suppressed proliferation of MCs. This inhibitory effect was partially reversed by the knockdown of E2A. In addition, the knockdown of FLASH induced cyclin-dependent kinase inhibitor p21WAF1/CIP1 (p21) expression, but did not affect p53 expression. Furthermore, overexpression of E12 and E47 induced p21, but not p53 in MCs, in the absence of FLASH. We also demonstrated that E2A and p21 expression at the peak of proliferation was followed by significant induction of FLASH in mesangial areas in rat proliferative glomerulonephritis. Moreover, we revealed that FLASH negatively regulates cellular senescence via the interaction with E12. We also demonstrated that FLASH is involved in the TNF-α-induced p21 expressions. These results suggest that the functional interaction of E2A and FLASH play an important role in cell proliferation and cellular senescence via regulation of p21 expression in experimental glomerulonephritis.

## Introduction

Excessive proliferation of cells and subsequent overproduction of cellular matrix contribute significantly to pathogenesis of various sclerotic diseases, such as liver cirrhosis, scleroderma, and renal failure. Mesangial cells (MCs) resemble smooth muscle cells and can modulate glomerular hemodynamics by controlling glomerular capillary surface area [1, 2]. A key feature of progressive glomerular diseases, such as lupus nephritis, diabetic nephropathy, and chronic glomerulonephritis, is abnormal growth and proliferation of MCs that are normally quiescent in mature animals. MC proliferation frequently precedes and is linked to an increase of the extracellular matrix in the mesangium and to glomerulosclerosis [3–5]. Intriguingly, some proliferative glomerulonephritis, such as acute glomerulonephritis and Henoch-Schӧnlein nephritis, are self-limiting. The limitation of proliferative potential in somatic cells imposed by cellular senescence has been proposed as a mechanism of tumor suppression [6, 7]. However, the details of the effects of cellular senescence in proliferative diseases in kidney are largely unknown.

The E2A transcription factor belongs to the basic helix-loop-helix (bHLH) family of proteins, which contains a conserved basic region responsible for DNA binding and a helix-loop-helix (HLH) domain for dimerization [8]. The E2A gene encodes two alternatively spliced products, E12 and E47, which differ only in their bHLH domains [9]. Both proteins modulate the expression of their target genes through the formation of homodimers or heterodimers with each other and heterodimers with other bHLH transcriptional factors, and are involved in the control of cellular-specific differentiation and cell proliferation [10, 11]. In particular, we recently demonstrated that E12 contributes to the progression of glomerulosclerosis via phenotypic change [12]. On the other hand, the four Id (inhibitor of differentiation) proteins (Id1, Id2, Id3, and Id4) dominantly dimerize and neutralize the transcriptional activity of bHLH proteins. It has been reported that Id1 mRNA is abundantly expressed in MCs [13]. Therefore, tight control of HLH levels and activity is necessary to prevent uncontrolled cell proliferation and dedifferentiation and may be necessary for tissue repair following injury. However, little is known about the precise mechanisms of control of bHLH transcription factors in MCs.

The eukaryotic cell cycle is tightly regulated through a precious balance of many regulatory components that exert their effects during the first gap phase (G1) of the cell cycle [14, 15]. Cell cycle-associated proteins, p16, p21WAF1/CIP1 (hereafter called p21), p27 and p53 are important in regulating the G1-S checkpoint in the cell cycle, and their functional alterations play key roles in cell proliferation and differentiation. As a proliferation inhibitor, p21 is presumed to play important roles for induction of cell-cycle arrest, which is considered to be transcriptionally activated by p53 [16]. In addition, p21 has been shown to regulate the MC proliferative response in mesangial proliferative glomerulonephritis [17]. Moreover, up-regulation of the p21 expression participates in the process of cellular senescence as well as DNA damage-induced cell cycle arrest in various tumor cell culture studies [18]. However, the role of p21 in the association between cell proliferation and cellular senescence in the context of proliferative glomerulonephritis remains unclear.

Cellular senescence can be defined as cell cycle arrest accompanying the exhaustion of replicative potential [19]. Senescence is a cellular stress response resulting in a blockade of cell proliferation, characteristic morphological changes, and the expression of characteristic molecular markers such as senescence-associated β-galactosidase (SA-β-Gal) [20, 21]. p53 has been generally known to be activated in response to a variety of cellular stress signals including DNA damage and triggers cell cycle arrest or apoptosis to prevent cells from undergoing transformation [22]. Moreover, p53 mediates the transcriptional activation of p21, which switches on the replicative senescence program [23].

Herein we identified caspase-8-binding protein FLICE-associated huge protein (FLASH) as a binding effector of E2A and examined the function of FLASH on cell proliferation by interacting with E2A. Moreover, we showed that knockdown of FLASH elicits cellular senescence. Furthermore, we found that FLASH is involved in TNF-α-induced p21 expression and thereby modulates the resolution of inflammatory kidney diseases.

## Materials and Methods

### Animals

Male Wistar rats (CLEA Japan, Inc., Japan) weighing 180–200 g were used in this study. Rats were housed under specific pathogen-free conditions. All animal experiments were performed in accordance with institutional guidelines, and the Review Board of Tokushima University granted ethical permission for this study. Experimental mesangial proliferative glomerulonephritis was induced by a single intravenous injection of anti-rat Thy-1 monoclonal antibody (1 mg/kg) (Cedarlane Laboratories, Ontario, Canada) as described elsewhere [24]. It has been reported that administration of the anti-Thy1 antibody results in an acute phase of complement-dependent MC lysis, followed by intense MC proliferation [25] and mesangial matrix accumulation [26]. The rats were sacrificed on days 0, 4 and 12 after the administration of anti-Thy-1 antibody. Age-matched rats were injected with vehicle only and were sacrificed as controls. Rats were euthanized by carbon dioxide inhalation. The number of each group was six. The whole-kidney protein extracts from Thy-1 rats or control rats (sham) were served for immunoprecipitation assay.

### Immunohistochemistry

Kidneys of Thy-1 nephritis rats and aged-match control rats were examined histopathologically. The kidneys were fixed in methyl Carnoy’s solution and embedded in paraffin for light microscopic examination and 2-μm-thick sections were stained with hematoxylin and eosin, periodic acid Schiff’s reagent, and periodic silver methenamine. Cryopreserved kidney tissues were cut in 5-μm-thick sections and fixed in methanol at 4°C for 15 min. To eliminate nonspecific staining, sections were incubated with the appropriate preimmune serum for 30 min at room temperature, followed by incubation with primary antibodies, anti-E2A, anti-FLASH, and anti-p21 (Santa Cruz Biotechnology, Santa Cruz, CA). The secondary antibody was an anti-rabbit antibody conjugated with FITC. Control kidney sections were incubated with a nonspecific rabbit IgG instead of the primary antibody.

### Cell culture

A glomerular MC line was established from glomeruli isolated from normal 4-week-old mice (C57BL/6JxSJL/J) and was identified according to the method described previously [27]. MCs were maintained in B medium (a 3:1 mixture of minimal essential medium / F12 modified with trace elements) supplemented with 1 mM glutamine, penicillin at 100 units/ml, streptomycin at 100 mg/ml, and 20% fetal calf serum. The cultured cells fulfilled the generally accepted criteria for glomerular MCs [27]. BeSO_4_•4H_2_O (BeSO_4_ in the text) was purchased from Sigma Aldrich; working solutions were freshly prepared in distilled water. Recombinant TNF-α was obtained from R&D Systems. The cell number was counted using a hemocytometer at the time points indicated.

### Cell proliferation assay

MCs were plated out at a low density in 96-well flat-bottomed microtiter plates in B medium/10% FCS. The next day, the siRNA for FLASH, E2A and the control (Invitrogen) were transfected. The proliferation of MCs was determined at 24, 48, and 72 hours after the siRNA transfections using a colorimetric immunoassay, based on the measurement of BrdU incorporation during DNA synthesis (Amersham Biosciences). The BrdU ELISA was performed according to the manufacturer's instructions.

### Yeast two-hybrid screening

Yeast two-hybrid screening was performed with a Matchmaker GAL4 two-hybrid system (Clontech, Palo Alto, CA) using the reporter *Saccharomyces cerevisiae* strain AH109 as described by the manufacturer. To generate a bait construct with the bHLH domain of E12 (505-651aa), the cDNA was amplified by PCR from the full-length mouse E12 cDNA (RIKEN, Japan), and inserted into the *Nco*I-*Pst*I site of the pGBKT7 vector. We prepared cDNA from mouse MCs and inserted it into the pGADT7-Rec vector. Primary screening was based on activation of the histidine selection marker by an interaction between bait and library proteins and was performed using histidine-negative plates. Secondary screening was based on further activation of a β-galactosidase reporter gene and was determined using blue/white colony screening. Library-derived plasmids from the candidate clones were rescued into the *E*. *coli* DH-5α and studied further.

### Expression plasmid construction

pME18S-FLAG-FLASH, which expresses the full-length mouse FLASH, was a kind gift from Dr. S. Yonehara (Kyoto University, Japan). The bHLH domain of E12 was digested from the bait construct with EcoRI sites, and cloned into the pCMV-Myc vector (Clontech). The full-length of mouse E12 was amplified by PCR from the full-length mouse E12 cDNA (RIKEN). The full-length and bHLH domain (504–649 aa) of mouse E47 were amplified by PCR from the cDNA in mouse MCs. They were cloned into the Sal I/Bgl II sites of PCMV-Myc vector. All constructs were sequence-verified, and mammalian expression constructs were evaluated by Western blotting.

### Transfection and co-immunoprecipitation

For the co-immunoprecipitation experiments, CHO-K1 cells were transfected using Fugene 6 (Roche) according to the manufacturer’s instructions. The cells were harvested 48 h after transfection, washed with PBS, and then lysed on ice in 50 mmol/L Tris-HCl (pH7.4), 150 mM NaCl, 1% NP-40, and 0.5% deoxycholate supplemented with a protease inhibitor cocktail (Roche) for 20 min. The lysate was centrifuged at 10,000 g for 20 min. Immunoprecipitation was performed by incubating whole cell lysate with anti-HA polyclonal antibody (Clontech) or anti-FLAG monoclonal antibody (Sigma) for 16 h, followed by incubating with protein G sepharose for 1 h. The immunoprecipitates were washed three times with sample buffer, resuspended in 2 × SDS sample buffer containing 2-mercaptoethanol, and boiled for 5 min. Samples were subjected to SDS-PAGE. Kidneys isolated from rats were minced and suspended in lysis buffer containing 150 mM NaCl, 10 mM Tris-HCl (pH 7.5), and 0.1% deoxycholate. The tissue suspension was homogenized, and the lysates were immunoprecipitated with anti-FLASH antibody and control immunoglobulin G. The presence of E2A and FLASH in the immunoprecipitates was determined by Western blotting with anti-E2A and anti-FLASH antibodies (Santa Cruz), respectively. Mouse anti-rabbit IgG (L27A9) monoclonal antibody (Cell Signaling Technology) was used as a secondary antibody to minimize masking produced by denatured heavy chains.

### Western blot analysis

Protein samples were fractionated on SDS-PAGE gels and electroblotted onto Hybond C extra nitrocellulose membrane (GE Healthcare) and then incubated with the primary antibodies. Myc-tagged monoclonal antibody (Clontech), anti-β-actin and anti-FLASH antibodies (SIGMA), anti-p15 and anti-p53 antibodies (abcam), and anti-E12, anti-E47, anti-p16, anti-p21, and anti-p27 antibodies (Santa Cruz Biotechnology) were used. Protein bands were visualized with the ECL Western blotting detection system (GE Healthcare).

### Small-interfering RNA

MCs (0.5 × 10^5^) were seeded into 12-well plates (Nunc) and grown until they were 60% to 80% confluent. The small-interfering RNAs (siRNAs) for E2A, FLASH and p53 (Dharmacon) or control scrambled siRNA (Dharmacon) were combined with Lipofectamine RNAi/MAX reagent (Invitrogen), and the cells were transfected according to the recommended protocol with siRNA (20 nM final concentration).

### Quantitative RT-PCR

Total RNA was isolated from mouse MCs using TRIzol (Invitrogen). Reverse-transcription was done using the SuperScript (Invitrogen) with oligo(dT) primer, followed by PCR. Quantitative expression analyses were performed using the MiniOpticon qPCR detection system (Bio-Rad). Normalization was performed using GAPDH as internal standards. TaqMan assay ID number used for PCR amplification for each gene transcript were listed in S1 Table.

### Detection of cellular senescence and senescence-associated gGalactosidase (SA-β-gal) activity assay

Cells were plated at 2.0 × 10^5^ in 6-well dishes. Cytochemical staining for SA-β-galactosidase was performed using a Senescence Cells Histochemical Staining Kit (Cell Signaling Technology) at pH 6.0. All the experiments were repeated three times, and one of the representative results is shown. Total MC cells and MCs exhibiting flattened and enlarged morphology were counted under bright field at× 100 magnification. SA-β-gal activities present in cell extracts were measured using a 96-Well Cellular Senescence Assay Kit (Cell Biolabs) according to the manufacturer's instructions. In addition, for morphological, cells were plated in 8-well chamber slides at a density of 1,000 cells per well under BeSO_4_ or TNF-αtreatment. Number of cells with or without morphological changes was counted in all fields and calculated morphologically changed cell percentages.

### Statistics

All data are expressed as means ± S.D. Significance was established using a paired two-tailed Student’s t-test. We also used unpaired Student's t-test and linear regression analysis, as appropriate. *P* values less than 0.05 were used as the criteria of statistically significant differences.

## Results

### Glomerular expression of E2A in experimental mesangial proliferative glomerulonephritis

**Fig 1:**
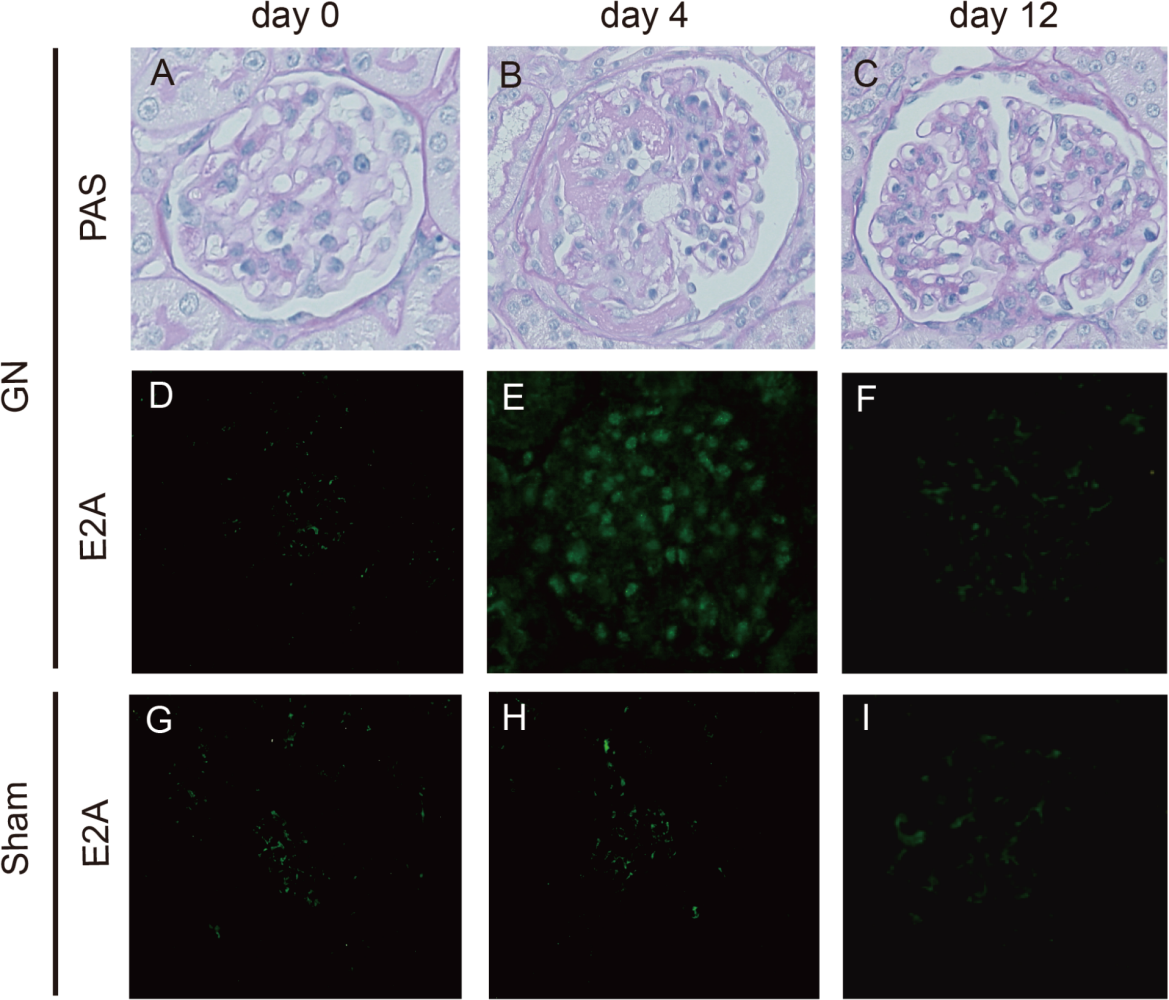
Induction of Glomerular E2A expression in Thy-1 nephritis rats. Representative light microphotograph from sections of the Thy-1 nephritis rat glomeruli stained with periodic acid-Schiff (PAS) stain and immunofluorescence analyses of E2A protein expression in Thy-1 glomerulonephritis (A-F) and sham-operated (Sham) control rats (G–I) at day 0 (A, D, and, G), at day 4 (B, E, and H) and at day 12 (C, F, and, I) (n = 6 in each group). Original magnification for all panels was ×400.

The regulatory molecules responsible for the coordination of differentiation and proliferation events in MCs are currently unknown. To elucidate the role of helix-loop-helix (HLH) transcription factors in MCs, glomerular expression of the ubiquitous HLH protein E2A was examined in Thy-1 glomerulonephritis rats. As previously shown, this rat model of mesangial proliferative glomerulonephritis, shows a peak the proliferation of MCs from day 4 through day 6, and subsides 12 days after the induction [5]. Thus, we performed indirect immunohistochemical studies at these points and found that E2A expression in glomeruli was indeed notably induced at day 4 in Thy-1 glomerulonephritis rats, and that the expression had returned to an almost normal level by day 12 (Fig 1). In contrast, these changes were not observed in the sham-operated control rats. These results suggest the possibility that E2A may be involved in the mechanisms of cell proliferation in glomeruli.

### E12 did not promote proliferation of MCs

**Fig 2:**
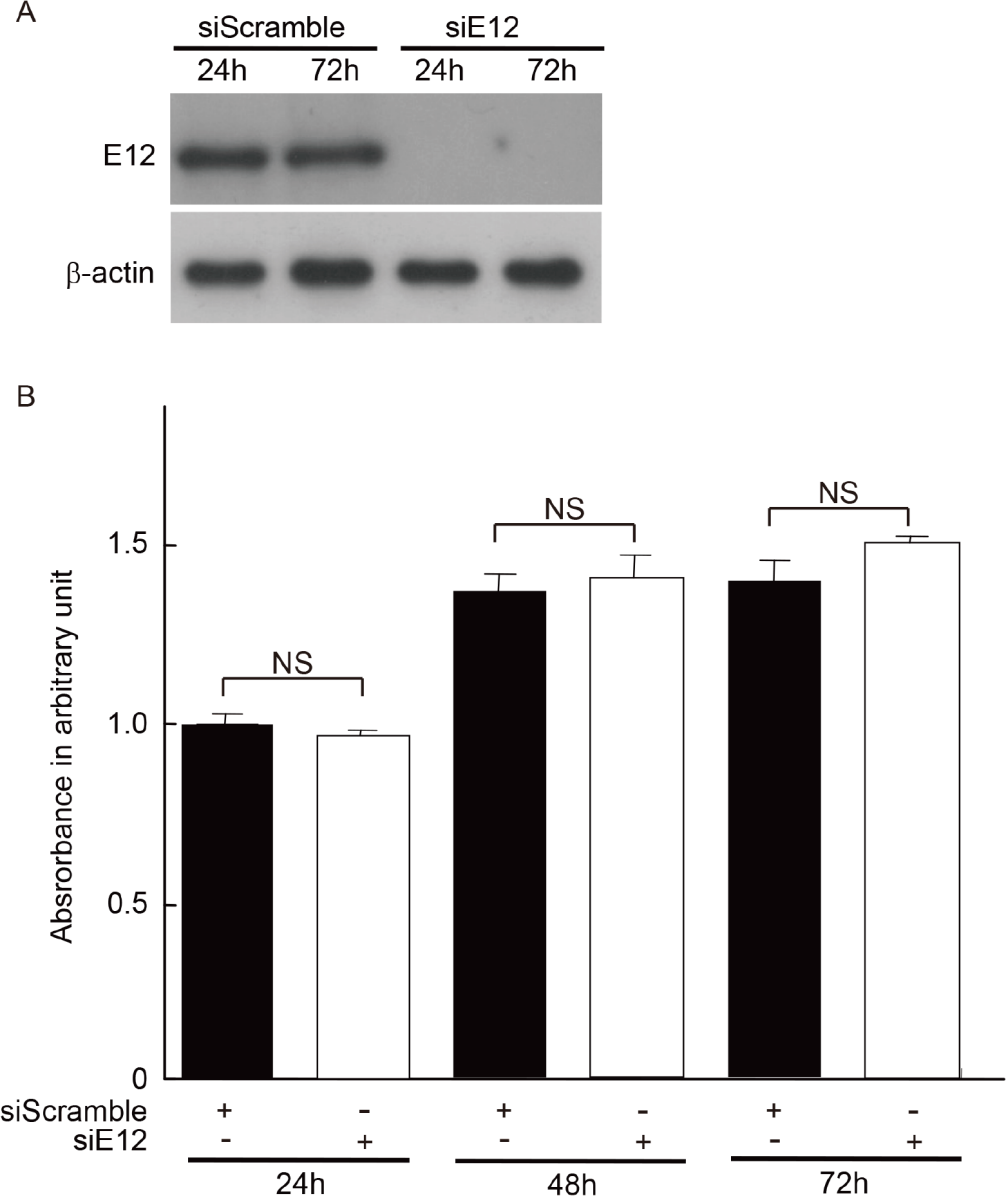
Association of cell proliferation and effect of siRNA-mediated E12 silencing on MCs. (A) Ablation of E12 expression following siRNA knockdown was confirmed by Western blot. One of three independent experiments is shown. (B) Effects of RNAi-mediated silencing of E2A on cell proliferation of MCs for the indicated times. The siRNA specific for E2A or the control siRNA (siScramble) was transfected into MCs. Results represent as arbitrary units and are shown as mean values ± SDs of at least five independent experiments. NS, not significant.

In our recent report, we demonstrated that E12 is involved in the progression of diabetic glomerulosclerosis via a phenotypic change in MCs [12]. Therefore, we investigated whether E12 directly influences cell proliferation in Thy-1 nephritis. Contrary to expectation, the knockdown of E2A by RNA interference did not affect BrdU incorporation (Fig 2A and 2B). From these results, we considered that a cofactor or cofactors likely modify the cell proliferation by interacting with E2A protein in MCs. Therefore, we used a yeast two-hybrid assay to identify the binding partners to E2A in MCs.

### Identification of FLASH as a novel E2A-interacting protein

**Fig 3:**
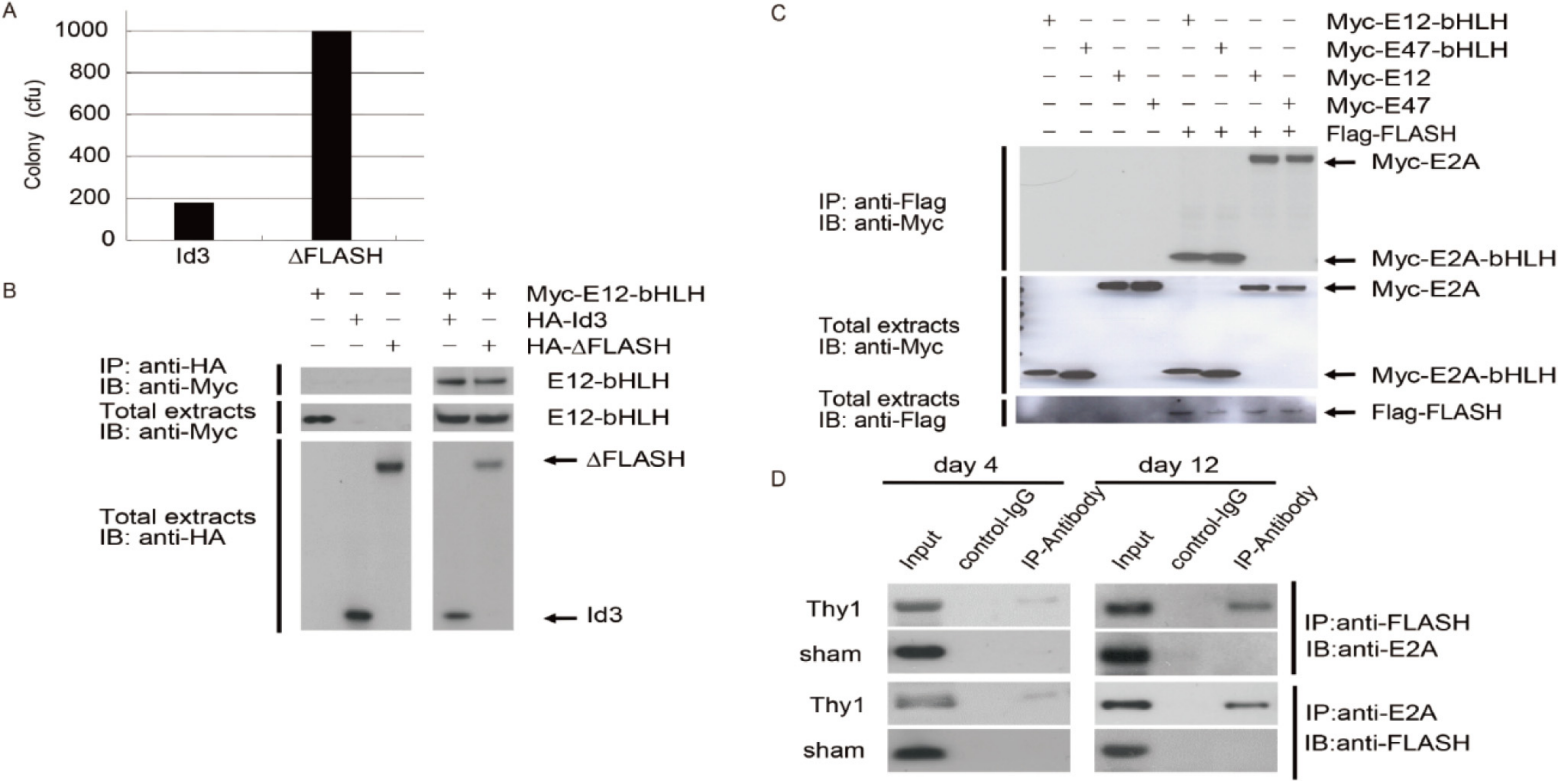
Physical interactions between E2A and FLASH. (**A**) Interaction between E12-bHLH and FLASH in a yeast two-hybrid assay. pGBKT7-E12-bHLH with pGADT7-Id3 (positive control) or pGADT7-FLASH-truncated (ΔFLASH) were transformed into AH109 as the manufacturer’s instructions. Colonies were counted and calculated as colony-forming units (cfus). (**B**) CHO-K1 cells were transfected with Myc-tagged E12-bHLH and HA-tagged Id3 or ΔFLASH. Cell lysates were immunoprecipitated (IP) with anti-HA antibody and immunoblotted (IB) with anti-Myc antibody (upper panel). Total cell lysates were blotted with anti-Myc antibody (middle panel) or anti-HA antibody (lower panel). One of three independent experiments is shown in each panel. (**C**) Co-immunoprecipitation of full-length FLASH and E12-bHLH E47-bHLH, full-length E12, or full-length E47 from co-transfected MC lysate was detected by immunoblot analysis. One of three independent experiments is shown in each panel. (**D**) Endogenous FLASH and E2A co-immunoprecipitation. Kidney lysates from Thy-1 glomerulonephritis or sham-operated (sham) rats at day 4 and day 12 were immunoprecipitated (IP). The presence of E2A and FLASH in the immunoprecipitates was determined by immunoblot (IB) with anti-E2A and anti-FLASH antibodies, respectively. (n = 3 in each group)

In the attempt to identify E2A-interacting proteins and clarify the role of bHLH transcription factors in MCs, we performed a yeast two-hybrid screening using the bHLH domain of E12 (E12-bHLH) as a bait to screen a cDNA library from mouse MCs. From 1.5 × 10^6^ independent transformants, three different kinds of clones were found to be true, interacting positives on selection medium SD/-His/-Leu/-Trp plate only when they were co-expressed with Gal4-E12-bHLH fusion protein. Positive two-hybrid protein interaction was verified by transfection of plasmids back into the host together with the original bait or with selected controls (Fig 3A). DNA sequence analysis revealed that the nucleotide sequence of one clone encodes a truncated caspase-8-binding protein FLICE-associated huge protein (CASP8AP2; FLASH). In order to confirm that E12-bHLH interacts with truncated-FLASH (ΔFLASH) in mammalian cells, HA-tagged ΔFLASH or a mock vector were overexpressed with Myc-tagged E12-bHLH in CHO-K1 cells. Cell lysates were immunoprecipitated by anti-HA antibody, and precipitates were immunoblotted with anti-Myc antibody. ΔFLASH could be precipitated with E12-bHLH, and a similar result was observed by well-known E2A-interacting protein Id3 (Fig 3B). Expression of E12-bHLH, ΔFLASH and Id3 were demonstrated by Western blots of total cell lysates used for immunoprecipitation (Fig 3B). In addition, the specific interaction of full-length of E2A and full-length of FLASH in MCs was confirmed in a co-immunoprecipitation assay. Myc-tagged E12-bHLH and Myc-tagged E47-bHLH were co-immunoprecipitated with Flag-FLASH only when both were co-expressed. Similarly, full-length of E12 and E47 were also co-immunoprecipitated with Flag-FLASH (Fig 3C). Furthermore, we demonstrated an association between endogenous FLASH and E2A in Thy-1 rat kidney at day 12 by co-immunoprecipitation using the anti-E2A antibody. However, this association was faintly observed at day 4 (Fig 3D and 3E). These results provide the evidence that the interaction between FLASH and E2A occurs in proliferative glomerulonephritis kidneys. Thus, collectively, we confirmed that E2A and FLASH have a specific physiological interaction.

### FLASH silencing inhibited proliferation of MCs

**Fig 4:**
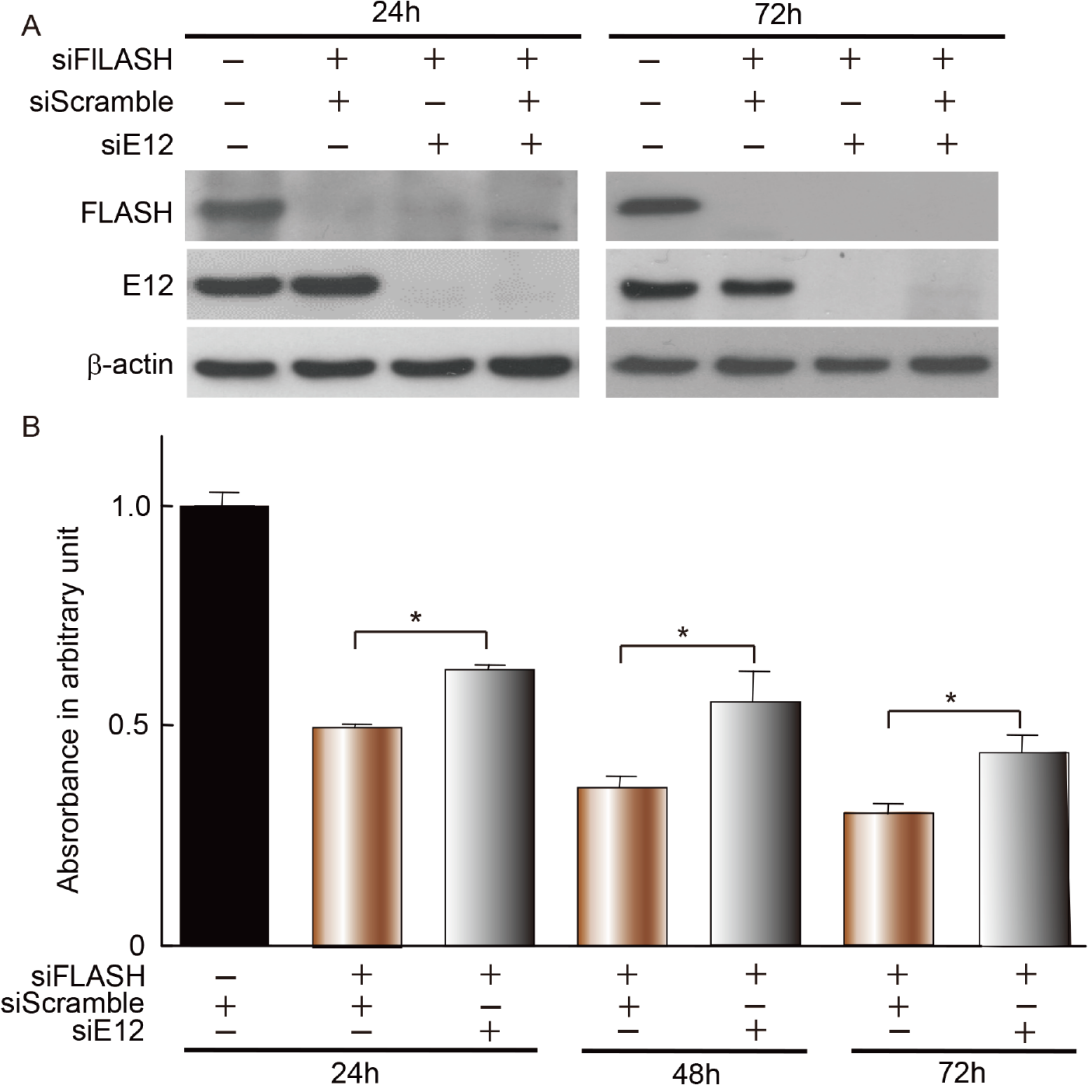
Inhibitory effect of siRNA-mediated FLASH silencing on MC proliferation. (**A**) Ablation of E12 and FLASH expression following siRNA knockdown was confirmed by Western blot. (**B**) Effects of RNAi-mediated silencing of E2A and FLASH on cell proliferation of MCs for the indicated times. The control siRNA (scrambled) or the siRNA specific for E2A alone, FLASH alone, and both E2A and FLASH was transfected into MCs. Data represent mean values ± SDs of at least five independent experiments. *P<0.01.

The next studies were conducted to determine whether FLASH is involved in cell proliferation by integrating with E12 in MCs. Although knockdown of E2A alone by RNA interference did not affect the BrdU incorporation (Fig 2B), knockdown of FLASH obviously inhibited the cell proliferation from 24 h through 72 h after transfection. Interestingly enough, the inhibitory effect of MCs by FLASH silencing at all points was significantly reversed when E2A was knocked down at the same time (Fig 4A and 4B). These results indicate that FLASH plays an important role in the proliferation of MCs through its interaction with E2A.

### FLASH affected cyclin-dependent kinase inhibitor p21^WAF1/CIP1^ expression in MCs

**Fig 5:**
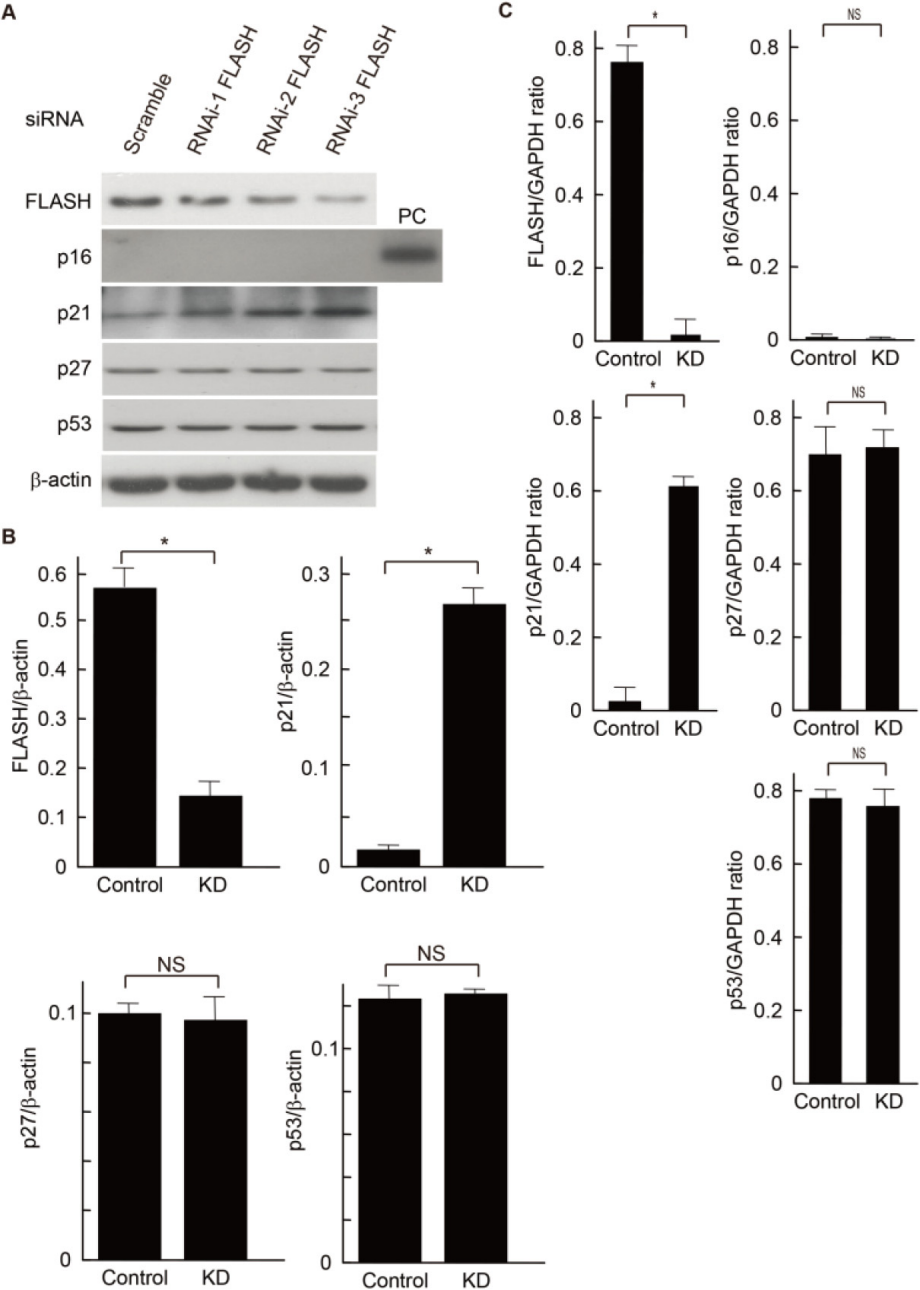
FLASH silencing with siRNAs affects p21 expression in MCs. Effects of RNAi-mediated silencing of FLASH in cultured mouse MCs. Scrambled siRNA (Scramble) was used as a control. (**A**) Equal amounts of cell lysates and total RNA were subjected to Western blot (**A**) and quantative RT-PCR (**C**), respectively. Since the MCs did not express p16 protein under the usual culture conditions, we have shown tha p16 expression data from p16-expressing MCs induced by cellular responses across multiple passage numbers. *PC*, positive control (late-passage mouse MCs). One of three independent experiments is shown in (**A**). (**B**) FLASH, p21, p27 and p53 protein levels were quantified by densitometric analysis and are expressed as the ratio between these proteins bands optical density and β-actin bands optical density. The quantitative comparison between control siRNA (scrambled) and the most effective siRNA specific for FLASH (RNAi-FLASH3 as KD) were analyzed. Results represent as arbitrary units and are shown as mean values ± SDs of at least three independent experiments. NS, not significant. *P<0.001. (**C**) Real-time RT-PCR showing relative mRNA levels for FLASH, p16, p21, p27 and p53. The quantitative comparison between scrambled siRNA (Control) and the most effective siRNA specific for FLASH (RNAi-FLASH3 as KD) were analyzed. Results represent as arbitrary units and are shown as mean values ± SDs of at least three independent experiments. NS, not significant. *P<0.001.

To clarify the role of FLASH for the regulation of cell proliferation, we investigated the protein expression levels of cell cycle-related molecules, such as p15, p16, p21, p27, and p53 after knockdown of FLASH. FLASH knockdown with RNA interference clearly increased both protein and RNA levels of p21, but did not affect the expression levels of other cell cycle-associated factors (Fig 5A, 5B and 5C). These results suggest that p21 is involved in the inhibitory effect of the coordination between FLASH and E2A in MCs.

### FLASH suppressed p21^WAF1/CIP1^ expression in correlation with E2A expression *in vivo* and *in vitro*

**Fig 6:**
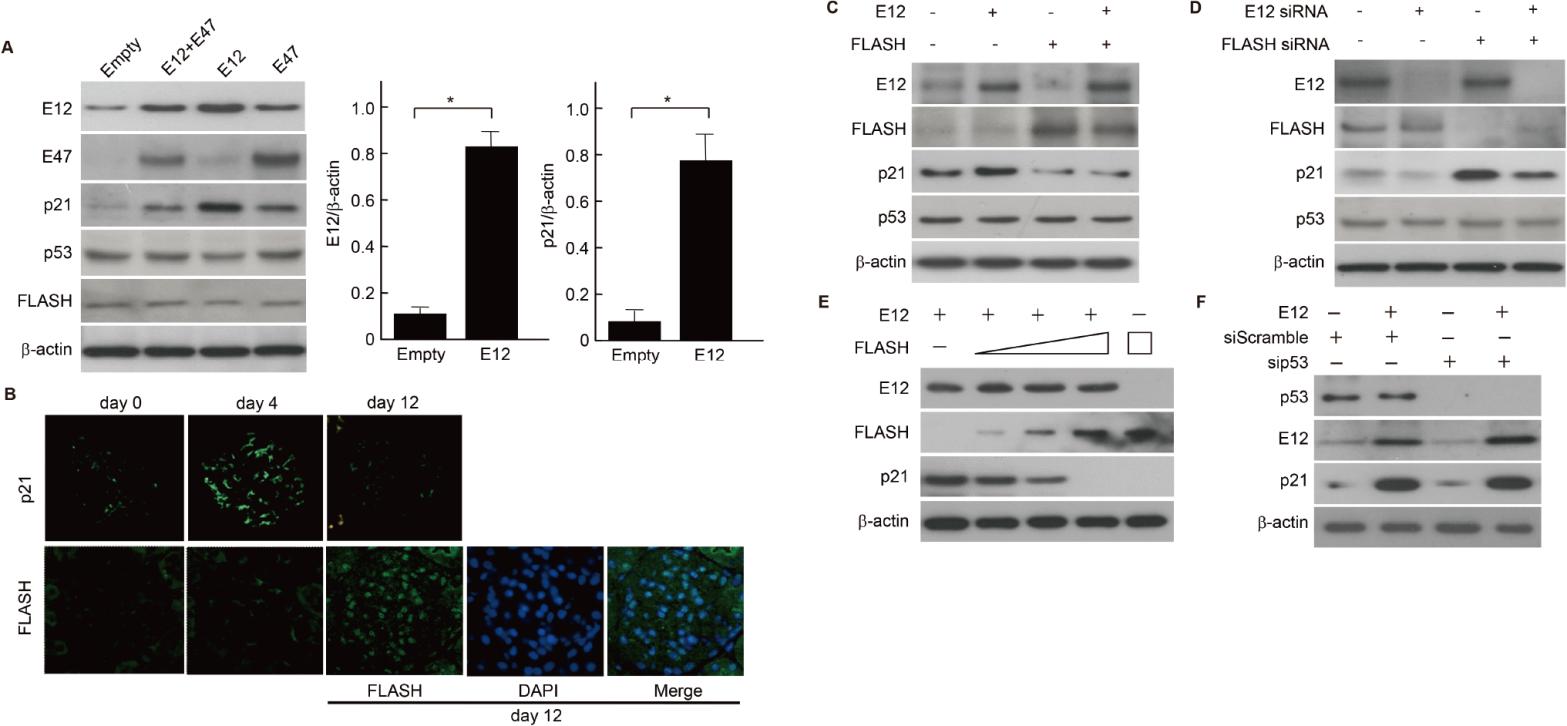
E12-induced p21 expression was attenuated by FLASH in MCs. (**A**) Western blot analyses of MCs transfected with full-length E12 and E47 plasmids, and empty vector (Mock). β-actin was used as an internal control. One of three independent experiments is shown. E12 and p21 protein levels were quantified by densitometric analysis and are expressed as the ratio between these proteins bands optical density and β-actin bands optical density. The quantitative comparison between the transfection of Empty vector (Empty) and the tranfection E12 expression vector (E12) were analyzed. Results represent as arbitrary units and are shown as mean values ± SDs of at least three independent experiments. *P<0.001. (**B**) Representative fluorescence staining pictures are shown. The induction of p21 in Thy-1 glomerulonephritis rats was followed by FLASH induction. The peaks of p21 and FLASH were observed at day 4 and day 12, respectively. FLASH expression in the glomeruli corresponded to the nuclei (DAPI in blue), and was shown in the merged image (Merge). Nuclei are illustrated by DAPI staining (blue). (**C**) Western blot confirmation of the attenuation of E12-induced p21 protein expression by the forced expression of FLASH. β-actin was used as an internal control. One of three independent experiments is shown. (**D**) FLASH silencing by specific siRNA clearly increased the protein level of p21 in MCs. β-actin was used as a loading control. One of three independent experiments is shown. (**E**) MCs were co-transfected in parallel with full-length E12 and increasing amounts of the FLASH expression plasmids (0.5, 1.5 and 3 μg), and transfected with FLASH expression plasmids alone (3 μg). For all transfection, the final concentration of DNA was adjusted to 5 μg using empty expression vector. Proteins were extracted and analyzed by Western blot analyses. β-actin was used as an internal control. One of three independent experiments is shown. (**F**) Silencing of p53 by specific siRNA clearly increased the protein level of p53 independetly of E12-induced p21expression in MCs. β-actin was used as a loading control. One of three independent experiments is shown.

To further elucidate the relationship between p21 and E2A, E2A was overexpressed in MCs by transient transfection of E12 and E47 expression vectors. Expression levels of p21 protein were increased along with the forced overexpression of E2A proteins, but no significant alteration was found in the FLASH and p53 proteins. In particular, the elevated p21 expression was paralleled with the level of E12 protein in MCs (Fig 6A). As E2A was increased at the peak of mesangial proliferation in Thy-1 glomerulonephritis rats (Fig 1), we further examined the glomerular expression level of FLASH and p21. Glomerular expression of p21 was also up-regulated in the mesangial area at the peak of mesangial proliferation (day 4). Although p21 expression was returned to the normal level, the increase in FLASH expression in glomeruli was followed at day 12 after induction of Thy-1 glomerulonephritis (Fig 6B). FLASH was localized in the nucleus coincident with the nuclear DAPI signal (Fig 6B). Next, we investigated whether the association between E12 and FLASH modulates p21 expression. The upregulation of p21 expression was dramatically attenuated in the presence of FLASH overexpression (Fig 6C). Likewise, the increased p21 expression was strongly upregulated by FLASH silencing by siRNA in mouse MCs (Fig 6D). These results imply that FLASH negatively regulates the E12-p21 axis during the resolution phase of proliferative glomerulonephritis. Moreover, to clarify the role of the interaction of FLASH and E12 for the expression of p21, we carried out overexpression of FLASH in mMCs. The protein expression levels of p21 was decreased in a dose-dependent manner of FLASH. In addition, the highest concentration of FLASH could suppress the expression of p21 without E12 expression vector transfection (Fig 6E). FLASH knockdown with RNA interference clearly increased both protein and RNA levels of p21, but did not affect the expression levels of other cell cycle-associated factors (Fig 5A and 5C). Because posttranscriptional modification involving phosphorylation of wild-type p53 has been proposed to be an important mechanism of p53 stabilization and functional regulation, we examined the role of p53 in the E12-p21 axis. Moreover, p53 activation depends on multiple sites by phosphorylation, acetylation, and sumoylation. Therefore, we perfomed p53 silencing by siRNA in mouse MCs and confirmed that p53 does not involve in the E12-p21 axis (Fig 6F).

### Role of FLASH and E12 in cellular senescence of MCs

**Fig 7:**
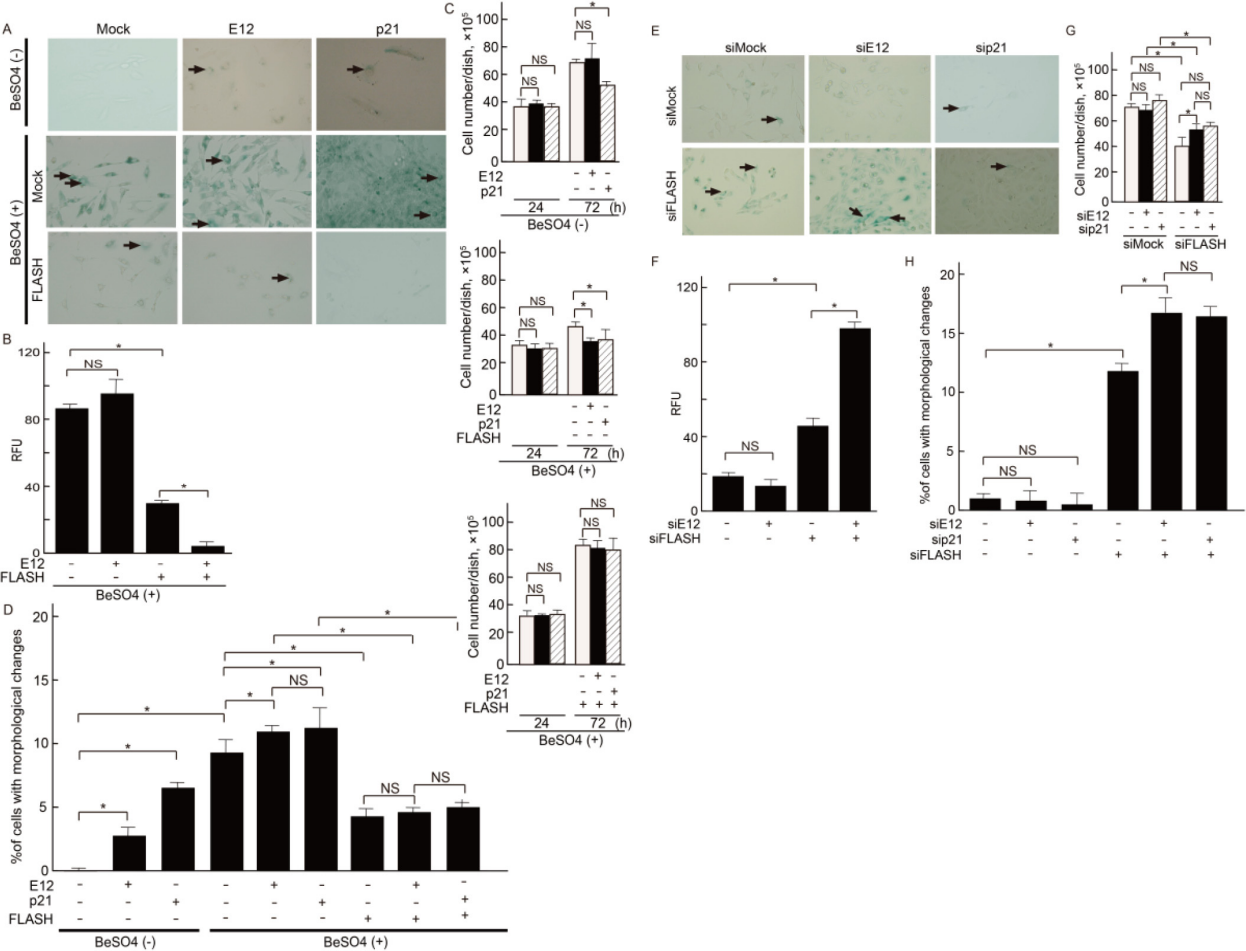
Effects of E12, p21 and FLASH on cellular senescence in MCs. (**A**) SA-β-gal staining at 48 h after transfection of expression plasmids for E12, p21 and FLASH, or empty vector (Mock) in MCs with 24 h-treatment with or without BeSO_4_ shows induction of senescence. Arrows show cells with morphological changes. (**B**) Cellular senescence by measuring SA-β-Gal activity using a fluorometric substrate. MCs with treatments as shown in (A) were lysed. Lysates were allowed to incubate with SA-β-Gal substrate for 1 hr at 37°C. Background-subtracted relative fluorescence units (RFUs) were normalized to optical density; excited at 360 nm, and showed emission at 465 nm. Experiments were conducted in triplicate (error bars are ± SDs). *P<0.001, NS, not significant. (**C**) Cell number was counted using a hemocytometer at the indicated conditions. Quantitative data for cell numbers from five independent experiments are presented as means ± S.D. *P<0.05. (**D**) Cells were counted on the basis of their morphological changes and expressed as the percentage of the total cell population. Results represent as arbitrary units and are shown as mean values ± SDs of at least three independent experiments. NS, not significant. *P<0.001. (**E**) SA-β-gal staining at 48 h after knockdown of E12, p21 and FLASH in MCs shows induction of senescence. Arrows show cells with morphological changes. (**F**) Cellular senescence by measuring SA-β-Gal activity. MCs with treatments as shown in (E) were lysed. RFUs were normalized to optical density; excited at 360 nm, and showed emission at 465 nm. Experiments were conducted in triplicate (error bars are ± S.D.). *P<0.001, NS, not significant. (**G**) The number of cells was determined microscopically using a hemocytometer at the indicated conditions. Quantitative data for cell numbers from five independent experiments are presented as means ± S.D. *P<0.05. (**H**) Cells were counted on the basis of their morphological changes and expressed as the percentage of the total cell population. Results represent as arbitrary units and are shown as mean values ± SDs of at least three independent experiments. NS, not significant. *P<0.001.

Cell cycle regulatory pathways mediated by CDK inhibitors play a critical role in senescence induction [28]. p21 is well-known to be involved in the regulation of not only cell proliferation but also cellular senescence in many cells. The p21 protein has been shown to inhibit apoptotic cell responses [29, 30]. Because growth arrest is a key step leading to cellular senescence, we first induced this change using beryllium salt (BeSO_4_) in cultured MCs. It has been reported that beryllium causes proliferation arrest with premature senescence and elicits expression of the p21 gene [31, 32]. To investigate whether FLASH and E12-p21 axis are involved in the control of cellular senescence in MCs, β-galactosidase (β-gal) activity was examined in mouse MCs. In the absence of BeSO_4_ stimulation, p21 overexpression showed a significant induction of cellular senescence. Moreover, Stronger β-gal staining was observed in the BeSO_4_-stimulated MCs, and the SA-β-gal activities present in cell extracts were enhanced in the presence of E12 or p21 overexpression. These staining were almost cancelled by forced FLASH expression (Fig 7A and 7B). Since cellular senescence causes phenotypic changes including an irreversible arrest of cell proliferation, the effects on cell proliferation by the interaction between FLASH and E12-p21 axis was investigated. Under BeSO_4_ stimulation, cell proliferation decreased with E12-p21 axis activation without FLASH overexpresssion. In contrast, FLASH overexpression clearly recovered the reduced cell proliferation (Fig 7C). Cultured senescent cells develop a distinct and recognizable flattened and enlarged morphology with which stains the perinuclear compartment blue. Senescent MCs exhibited the characteristic morphology in E12-p21 axis activation, but the morphological changes were suppressed by FLASH expression (Fig 7D). To examine the link between cell proliferation and cellular senescence under the inhibitory effects of FLASH, a knockdown assay was performed as shown in Fig 4. Although knockdown of E12 alone or p21 alone by RNA interference did not affect the cellular senescence, knockdown of FLASH strongly led MCs to cellular senescence (Fig 7E). In addition, the SA-β-gal activities present in cell extracts by knockdown of FLASH expression were enhanced (Fig 7F). Cell proliferation decreased with FLASH silencing in mouse MCs (Fig 7G). In contrast, FLASH overexpression clearly recovered the reduced cell proliferation (Fig 7C). Consistent with these data, the MCs with silencing of FLASH exhibiting significantly more flattened and more enlarged morphological changes compared with control (Fig 7H).

### FLASH is involved in TNF-α-induced p21 expression in MCs

**Fig 8:**
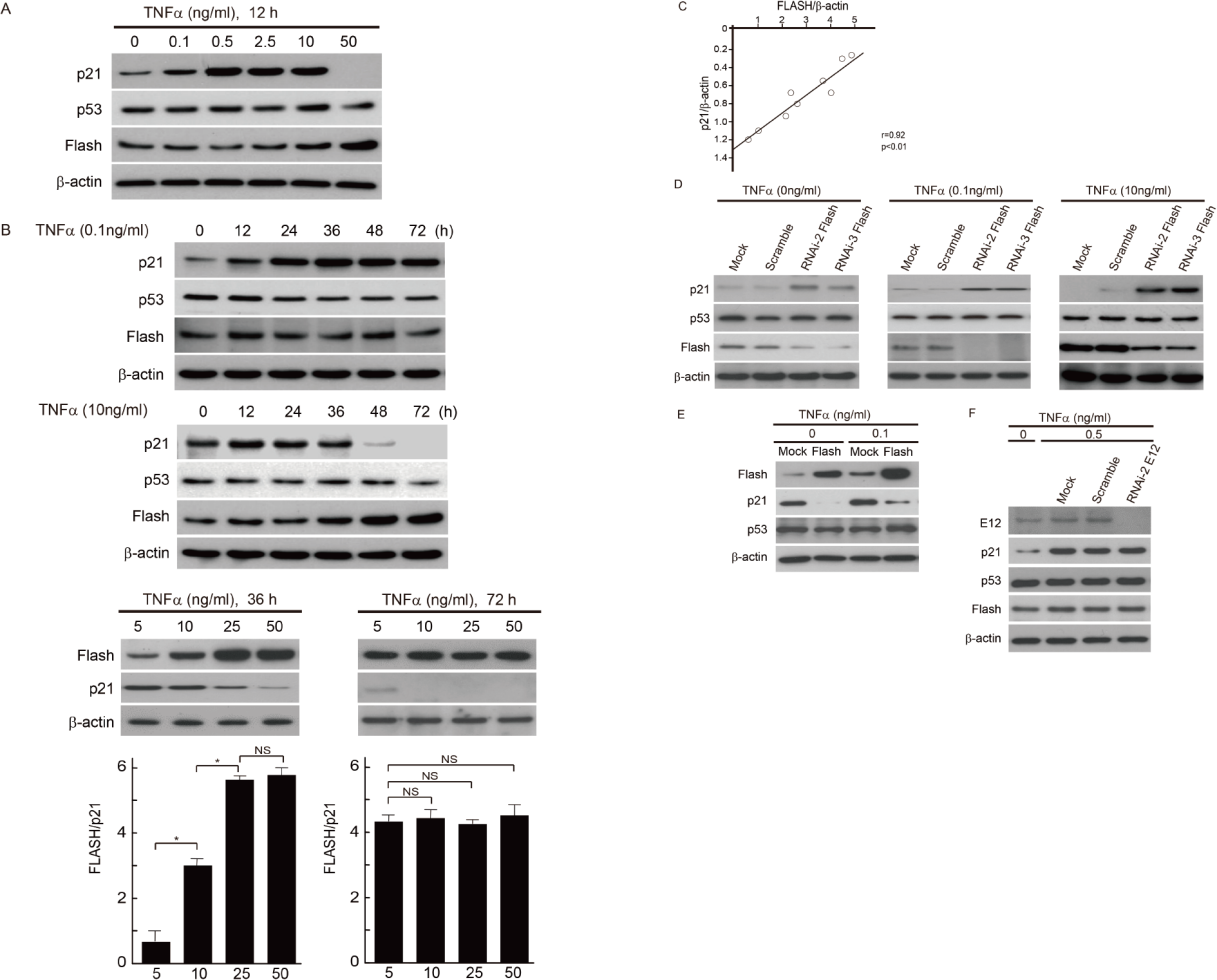
Negative regulation of TNF-α-mediated p21 expression through upregulation of FLASH in MCs. (A) Western blot analyses of MCs exposed to TNF-α at the indicated concentrations for 12 h. β-actin was used as an internal control. One of three independent experiments is shown. (B) Time course of p21, p53 and FLASH expression levels in MCs treated with low (0.1 ng/ml) or high-dose (10 ng/ml) TNF-α. In addition, the effects of comparative high concentration of TNF-α on p21 expression in a dose-dependent manner for 36 and 72 h stimulation were monitored by Western blot analyses. One of three independent experiments is shown in each panel. β-actin was used as an internal control. FLASH and p21 protein levels were quantified by densitometric analysis and are expressed as the ratio between these proteins bands optical density. Results represent as arbitrary units and are shown as mean values ± SDs. NS, not significant. *P<0.001. (C) Regression analysis comparing the changes in p21 levels with changes in FLASH expression levels under high concentration of TNF-α stimulation, the expression level of p21 negatively correlated with the expression level of FLASH (R = 0.922; P < 0.01). (D) MCs were treated with only transfection reagent (Mock) or transfected with scrambled or FLASH siRNA. After the siRNA transfection and successive 24 h incubation, MCs were treated with low concentrations of TNF-α (0 or 0.1 ng/ml) for 12 h, and treated with high concentrations of TNF-α(10 ng/ml) for 48h, respectively. After these stimulation of TNF-α, the cells were harvested and subjected to western blot. One of three independent experiments is shown. (E) Western blot analyses of MCs transfected with full-length FLASH plasmid, and empty vector (Mock) under low concentration of TNF-α. β-actin was used as an internal control. One of three independent experiments is shown. (F) MCs were mock-transfected (Mock) or transfected with scrambled or E12 siRNA. After the siRNA transfection and successive 24 h incubation, MCs were treated with 0 or 0.5 ng/ml of TNF-α. β-actin was used as an internal control. One of three independent experiments is shown.

**Fig 9:**
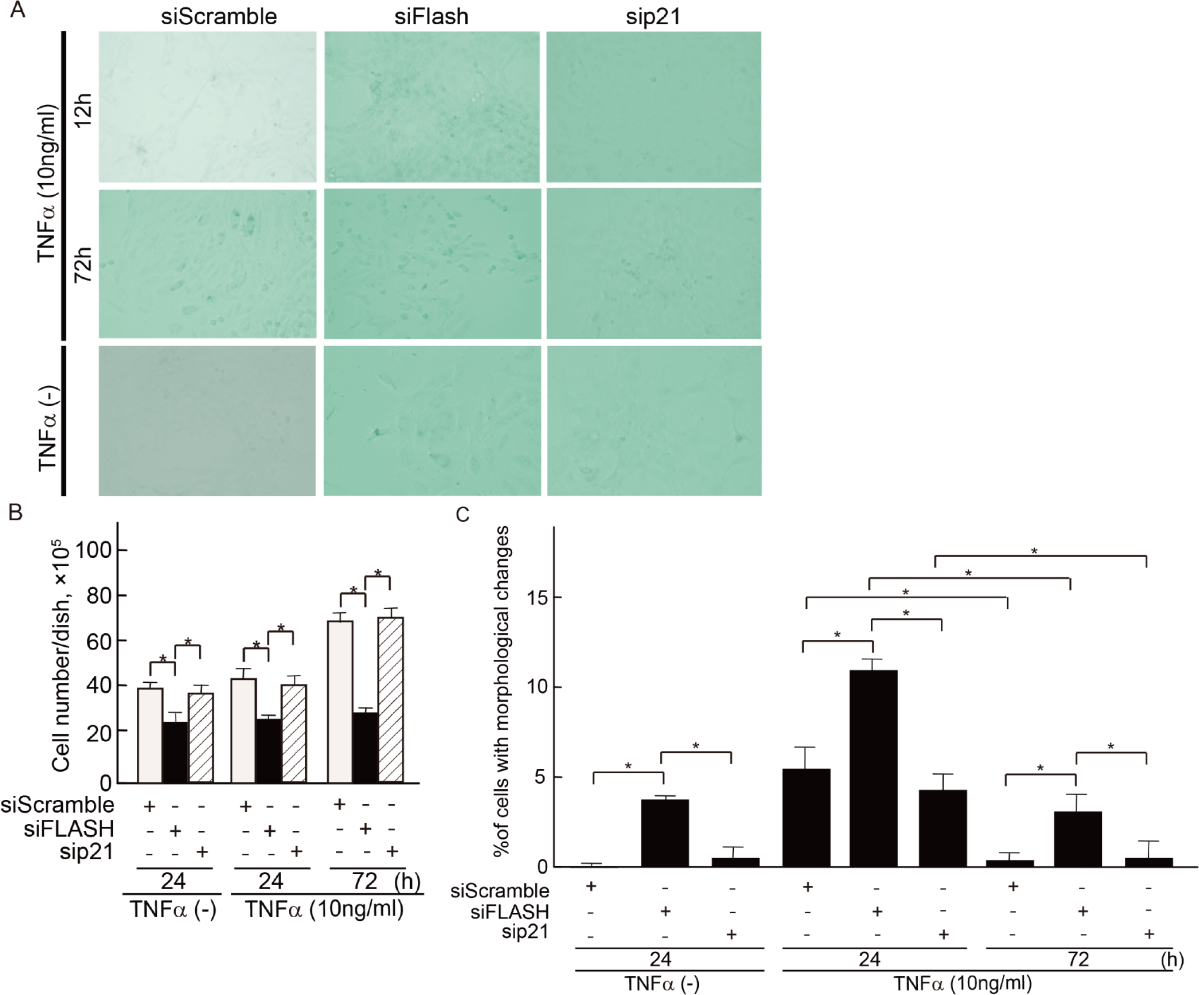
Effects of p21 and FLASH under TNF-α stimulation on cellular senescence in MCs. (**A**) SA-β-gal staining at 48 h after knockdown of p21 and FLASH in MCs in the absence or presence of TNF-α shows induction of senescence. Representative data from one out of five independent experiments are shown. (**B**) The number of cells was determined microscopically using a hemocytometer at the indicated conditions. (**C**) Cells were counted on the basis of their morphological changes and expressed as the percentage of the total cell population. Results represent as arbitrary units and are shown as mean values ± SDs of at least three independent experiments. *P<0.001.

The timely induction of apoptosis is critical for inflammation resolution, and it is thought that the acceleration of apoptosis may facilitate resolution at inflammatory sites [33, 34]. TNF-α is widely involved in multifunctional signaling pathways in inflammation and apoptosis. To address whether TNF-α affects expression of FLASH in rat glomerulonephritis, we monitored expression of FLASH protein in mouse MCs treated with TNF-α. Although low doses (0–0.5 ng/ml) of TNF-α stimulated expression of p21 in a dose-dependent manner, high doses (more than 2.5 ng/ml) of TNF-α did not induce p21 anymore, and interestingly, p21 expression was shut off by TNF-α at 50 ng/ml. At this highest concentration, TNF-α strongly induced FLASH expression (Fig 8A). Next we monitored time-dependent expressions of p21 and FLASH in MCs treated with a low- or high-dose TNF-α. Low-dose TNF-α treatment promoted induction of p21 expression for the first 24 hours with no significant induction of FLASH expression. In contrast, induction of p21 expression was suppressed in MCs treated with high-dose of TNF-α, which was inversely correlated with significant induction of FLASH expression. Moreoer, the longer expose (72 h) to high-dose of TNF-α showed almost complete suppression of p21 expression (Fig 8B). Regression analysis showed p21 expression significantly negatively correlated with FLASH expression in mMCs treated with high concentrions of TNF-α (Fig 8C). These findings imply that FLASH is involved in the negative regulation of TNF-α-p21 signaling axis. Therefore, we examined the effects of knockdown of FLASH on TNF-α-induced p21 expression. The effect of low and high concentration levels of TNF-α stimulation on p21 expression was dramatically cancelled by FLASH silencing in mouse MCs. Similar results were observed in mMCs without TNF-α stimulation (Fig 8D). Moreover, we performed FLASH overexpression assay in mMCs, suggesting that FLASH crucially suppresses the TNF-α-induced p21 expression in the setting of low concentration of TNF-α (Fig 8E). Furthermore, we investigated the role of E12 for TNF-α-induced p21 expression. Under the condition that FLASH was not induced, E12 was not involved in the induction of p21 (Fig 8F). In summary, upregulated FLASH expression plays a central role in the suppressive effects of the TNF-α-induced p21 expression. In respect to cellular senescence in MCs treated with TNF-α, we investigated senescent phenotypes in the absence or presence of TNF-α. As expected, knockdown of FLASH enhanced the senescent phenotypes in each conditions (Fig 9A, 9B and 9C).

## Discussion

Excessive proliferation of cells contributes significantly to the pathogenesis of several diseases, including cancer, atherosclerosis, psoriasis, scleroderma, and glomerulonephritis. Proliferative glomerulonephritis is primarily characterized by increased cellularity in the mesangial area. Proliferation of MCs is a common histologic feature in many glomerular diseases and is a critical process in progressive glomerular sclerosis [35, 36]. In contrast, it is generally known that some proliferative glomerulonephritis are self-limiting. However, molecular mechanisms underlying the process to regain the homeostasis after cell proliferative changes are still undetermined. The bHLH proteins are a superfamily of transcription factors that have been well characterized in mammalian systems and are important regulatory components in transcriptional networks controlling a diversity of processes from cell proliferation to cell lineage establishment. Even in MCs, it has been considered that bHLH transcription factors play crucial roles not only in cell differentiation but also in cell proliferation. However, no MC-specific bHLH protein has been reported so far. Here, we report a regulatory mechanism of bHLH proteins and a novel bHLH-binding protein in proliferative changes of MC in glomerulonephritis. During kidney development, E2A is diffusely expressed throughout the kidney in the glomeruli, tubules and interstitial cells; at maturity, its expression is reduced [37]. However, little is known about the role of E2A in proliferative glomerulonephritis. E2A is ubiquitously expressed in most tissues, and the most notable feature of the E2A expression is its high levels of expression in some areas of rapid cell proliferation [38]. Therefore, we first investigated the glomerular expression of E2A in Thy-1 glomerulonephritis rats, which are a good-characterized model whose morphological features closely resemble those of human mesangial proliferative glomerulonephritis [5, 39]. E2A expression was clearly increased in the mesangial proliferative phase, suggesting that E2A plays a part in the early stage of proliferative changes of MCs. Despite the importance of E2A in regulation of cell proliferation and in normal kidney development, knockdown of E2A did not affect the proliferation of MCs. Therefore, to identify the partner of the E2A protein, we performed a yeast two-hybrid screening in MCs.

As a result, we identified FLASH, also termed CASP8AP2, as a novel E2A-binding partner, and the interaction was confirmed by *in vitro* and *in vivo* assays. FLASH was originally identified as a component of the apoptosis-signaling complex known as the death-inducing signaling complex, which is assembled in response to Fas ligand binding [40]. Recent reports have shown that FLASH is a nuclear protein, is mainly localized in Cajal bodies, and plays an important role in histone transcription and S-phase progression [41]. FLASH was also reported to be essential for cell division by high-throughput screening in a genome-wide analysis using short interfering RNAs [42]. On the other hand, Pagliuca et al. reported that ectopic expression of E2A in the kidney embryonic cell line 293T causes an increase in the S-phase fraction [43]. In contrast, there have been some papers showing that ectopic expression of E2A appears to cause G_1_ arrest and suppresses cell proliferation in T-cell lymphomas and NIH 3T3 fibroblasts [44, 45]. Therefore, it is likely that there are different regulatory mechanisms for E2A function in different cells. In the present study, knockdown of E2A alone did not alter cell proliferation in MCs, whereas the knockdown of FLASH by RNA interference suppressed cell proliferation. The inhibitory effect was significantly attenuated by combination with knockdown of E2A by RNA interference. These results suggest that FLASH plays an important role in cell proliferation of MCs via its interaction with E2A.

Cell proliferation is governed by the eukaryotic cell cycle [46], which is delicately regulated by a variety of signals that act to inhibit cell cycle progression. Interestingly, in this study, we have demonstrated that FLASH negatively regulated the expression of the CDK inhibitor, p21. E2A is well known to increase expression of the p21 cyclin-dependent kinase inhibitor and inhibit cell cycle progression [47]. As expected, E2A upregulated the expression of p21 in MCs. At the peak of MC proliferation in Thy-1 glomerulonephritis (day 4), it would appear that the expression of p21 was upregulated to suppress MC proliferation, and the increase in E2A expression seemed to assist the expression of p21. In addition, it was speculated that FLASH is upregulated in order to suppress E2A. In fact, FLASH was additionally upregulated 12 days after the induction of Thy-1 glomerulonephritis, such that the expressions of E12 and p21 were returned to their normal levels. For example, in tumor suppression, the arrest of cell proliferation is an important feature of replicative senescence. However, in addition to an irreversible growth arrest, senescent cells display other phenotypic changes, such as the resistance to apoptotic stimuli. Depletion of FLASH was reported to halts growth by down-regulating histone biosynthesis and arrests the cell cycle in S-phase [41]. Depletion of FLASH was also known to reduce intracellular levels of the anti-apoptotic proteins. Therefore, further investigations are necessary to clarify the detailed molecular mechanisms regarding the interaction between cellular senescence and apoptosis modulated by FLASH in glomerulonephritis.

A lot of evidence suggests that p21 can be induced by p53 in response to DNA damage in a variety of cells and is necessary for the p53-mediated G1 arrest, providing a tight security network toward tumor suppression [48–51]. Moreover, p21 has been reported to be a critical target of p53 during senescence [52]. In this study, however, both overexpression and knockdown of FLASH affected the expression levels of p21 independently of p53, implying that p53 is not a major regulator of p21 gene expression in the MCs. A recent report demonstrated that p21 plays a survival role against apoptosis and induces irreversible senescence in a manner independent of p53 in human cancer cell lines [53]. Therefore, these results suggest that p21 may be a key determinant of the resolution in proliferative glomerulonephritis.

Effective resolution of inflammation requires cessation of proliferative changes in the process of glomerulonephritis. In particular, TNF-α plays a critical role in both tissue destruction and damage recovery, maintaining the reversibility of microenvironments, modulating cellular changes, and tissue remodeling in the inflammatory responses [54]. Moreover, some recent reports have heightened the awareness that resolution is an active process which requires activation of endogenous programs that enable the host tissue to maintain homeostasis [55, 56]. We here revealed that the functional interaction of E2A and FLASH play an important role in cell proliferation of MCs via the regulation of TNF-α-p21 expression. Since p21 was discovered as a senescent cell-derived inhibitor that can mediate cellular senescence [29], the interaction of FLASH and E2A-p21 axis may play a pivotal role in cell proliferation and cellular senescence in glomerulonephritis. Collectively, these results indicate that induction of p21 and FLASH in glomeruli may lead to the resolution of the proliferative changes and subsequent renal tissue homeostasis, independently of p53. However, the intraglomerular concentration of TNF-α would be important to regulate the resolution process. Therapeutic approaches for irreversibly progressive glomerular diseases are currently limited to supportive therapy to slow the loss of function of kidney. Our findings offer insights into the nature of the resolution of proliferative diseases and may provide potential therapeutic targets for inhibiting the progression of various renal diseases leading to sclerosis by suppressing the pathologically in the activated MCs.

## Supporting Information

S1 Table TaqMan assay ID number used for PCR amplification in qPCR in Fig 5. (DOCX)
