# Supplementary material for: A Novel Interaction between FLICE-Associated Huge Protein (FLASH) and E2A Regulates Cell Proliferation and Cellular Senescence via Tumor Necrosis Factor (TNF)-Alpha-p21WAF1/CIP1 Axis
Source: PLoS One. 2015 Jul 24;10(7):e0133205. doi: 10.1371/journal.pone.0133205 (PMC4514670; doi:10.1371/journal.pone.0133205)
Supplement: S1 Table — (DOCX) [file pone.0133205.s001.docx]

Table S1. TaqMan assay ID number used for PCR ampliﬁcation in qPCR in Figure 5

| Target gene | TaqMan assay ID number (Applied Biosystems) |
| --- | --- |
| GAPDH | Mm99999915_g1 |
| Flash | Mm00516278_m1 |
| p16 | Mm00494449_m1 |
| p21 | Mm04205640_g1 |
| p27 | Mm00438168_m1 |
| p53 | Mm01731290_g1 |
